# Application of Fucoxanthin‐Loaded Probiotic Membrane Vesicles in Dietary Intervention of High‐Fat Diet Induced Obese Mice and Color Improvement for Fruit Juice

**DOI:** 10.1002/fsn3.71686

**Published:** 2026-03-23

**Authors:** Duo Liang, Yueling Sun, Jinfeng Wu, Ziling Liu, Rong Lin, Ritian Jin, Shen Yang

**Affiliations:** ^1^ College of Ocean Food and Biological Engineering Jimei University Xiamen Fujian China

**Keywords:** anti‐obesity, delivery system, fucoxanthin, membrane vesicles, natural colorant

## Abstract

Development of nano‐encapsulated bioactive compounds as nutritional interventions and food‐grade additives presents a critical pathway for advancing translational applications in functional food development and health sciences. This study validates the dual efficacy of fucoxanthin‐loaded probiotic membrane vesicles (FX‐MVs) as both a nutritional intervention for high‐fat diet‐induced obesity and a natural pigment for food applications. The results demonstrated that FX‐MVs significantly suppressed body weight gain, decreased the ratio of white adipose tissue, and reduced serum and hepatic lipid levels in mice. Meanwhile, FX‐MVs reshaped the gut microbiota composition and enhanced microbial homeostasis. Furthermore, FX‐MVs application markedly improved the color attributes of fresh apple juice and preserved its original flavor profile, without introducing undesirable olfactory or gustatory notes. This work provided novel insights into fucoxanthin‐loaded probiotic membrane vesicles as effective nutrition components in obesity intervention and natural colorant in food systems, demonstrating synergistic potential in both nutraceutical and technological applications.

## Graphical abstract

Dual‐functional fucoxanthin delivery using probiotic membrane vesicles for obesity intervention and fruit juice color enhancement.

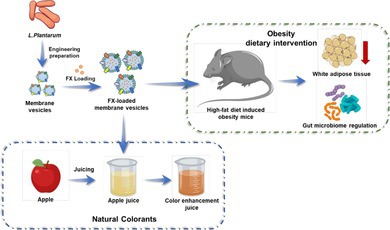

## 1 Introduction

Since the mid‐1970s, the prevalence of obesity has tripled due to common environmental factors, representing a global health threat that affects people of all ages and genders (Heymsfield Steven and Wadden Thomas 2017; Kumanyika and Dietz William 2020). Overabundant evidence showed obesity not only affected individuals' activity and psychology levels, but was also associated with hypertension, cardiovascular diseases, diabetes, and chronic diseases development (Boutari et al. 2023; Krzysztoszek et al. 2021; Parto and Lavie 2017; Romain et al. 2019; Seravalle and Grassi 2017). The most direct factor that leads to obesity is the high‐fat diet (HFD). HFD triggers obesity through multiple mechanisms, including increasing intestinal lipid absorption, disrupting adipose and hepatic lipid metabolism, and modulating energy metabolism in adipose tissue (Tang et al. 2024). Although pharmacotherapy is an essential part of treating obesity, it should be mentioned that new therapeutic methods including gene‐directed therapy, targeted therapy and fecal transplantation are also promising in obesity treatment (Gadde et al. 2018; Jimenez et al. 2018). Nonetheless, the high cost and safety issue limit their application to public obesity treatment on a large scale. Hence, lifestyle modification and nutritional intervention represent healthier and more appropriate strategies for the general population to manage obesity (Wadden et al. 2012).

As a prolific source of biological resources, the ocean offers abundant marine raw materials and effective nutritional interventions for obesity management through regular dietary intake. Certain marine bioactive compounds—such as peptides, polyphenols, and functional lipids—can play an effective role in daily diets to combat obesity (Kar et al. 2024; Man et al. 2023; Pereira and Cotas 2023). Fucoxanthin (FX) is a marine carotenoid existing in the macroalgae and microalgae, which has recently attracted considerable research interest in the nutrition and health fields because of its putative anti‐obesity, anti‐inflammation, anti‐cancer and eye‐protective effects (Chiang et al. 2020; Li, Ren, et al. 2020; Li, Xu, et al. 2020; Miyashita and Hosokawa 2017; Terasaki et al. 2020). Prior studies have shown that FX may play an important role in combating obesity by blocking adipocyte differentiation, regulating the expression of proteins involved in adipogenesis and glucose uptake, and ameliorating gut microbiota dysbiosis (Koo et al. 2019; Miyashita and Hosokawa 2017; Sun et al. 2020). However, the utilization of FX in the food industry is still severely constrained owing to its instability in air, high temperatures, and acidic conditions, along with its poor water solubility and highly unsaturated characteristics (Yusof et al. 2022). These challenges can be addressed by developing FX delivery systems, which enhance the stability, release, bioavailability, and bioactivity of FX. At present, the main delivery systems of FX are nanoparticles, microcapsules, emulsion, gel, coacervate, nanofiber and so on, and the material of the carrier includes protein, polysaccharide, lipid etc. (Chen et al. 2022; Tie and Tan 2022; Yuan et al. 2023). The development of various delivery systems can offer versatile options for the application of FX across various food products, thereby enhancing the commercial applicability of FX.

Food colorants are extensively utilized in the food industry to enhance the visual appeal and marketability of products during processing, storage, and packaging. They provide a distinctive appearance that can influence consumer preferences. However, the primary function of current artificial colorants is to boost sales rather than contribute any significant nutritional value (de Oliveira et al. 2024). The utilization of natural colorants, which possess nutritional and pharmacological benefits, to replace artificial colorants represents a promising direction for the future food industry. Natural colorants offer a broad spectrum of hues applicable in food products, such as red, green, blue‐violet, and orange‐yellow (Liang et al. 2025). However, these pigments are typically fat‐soluble, exhibiting poor water solubility and low bioavailability. This characteristic poses a significant challenge for their incorporation into most food matrices, thereby limiting the application of various natural pigments as effective natural color enhancers in food products. Even though extensive research efforts have been made to enhance the water solubility and bioavailability of these natural pigments using various delivery systems, evidence of their practical application in food systems remains scarce.

Compared with traditional delivery systems (e.g., nanoparticles and emulsions), membrane vesicles derived from probiotics have emerged as a novel delivery platform, exhibiting multidimensional advantages in encapsulation and functionalized delivery. Although traditional delivery carriers can enhance the stability and bioavailability of fucoxanthin, they still face bottlenecks such as insufficient biocompatibility, limited intestinal targeting ability, and a single functional dimension. In contrast, membrane vesicles derived from food‐grade probiotics not only retain the low immunogenicity and excellent biocompatibility of the natural membrane structure, but also possess potential intestinal epithelial adhesion and microbiota regulation capabilities due to their probiotic membrane properties (Dong et al. 2023). Moreover, membrane vesicles can effectively preserve the stability of bioactive components under various adverse conditions, indicating their strong application potential in complex food matrices (Alves et al. 2016). Therefore, probiotic‐derived membrane vesicles not only offer a safe and efficient delivery strategy for natural colorants, but also open up new avenues for their multifunctional applications in food.

**FIGURE 1:**
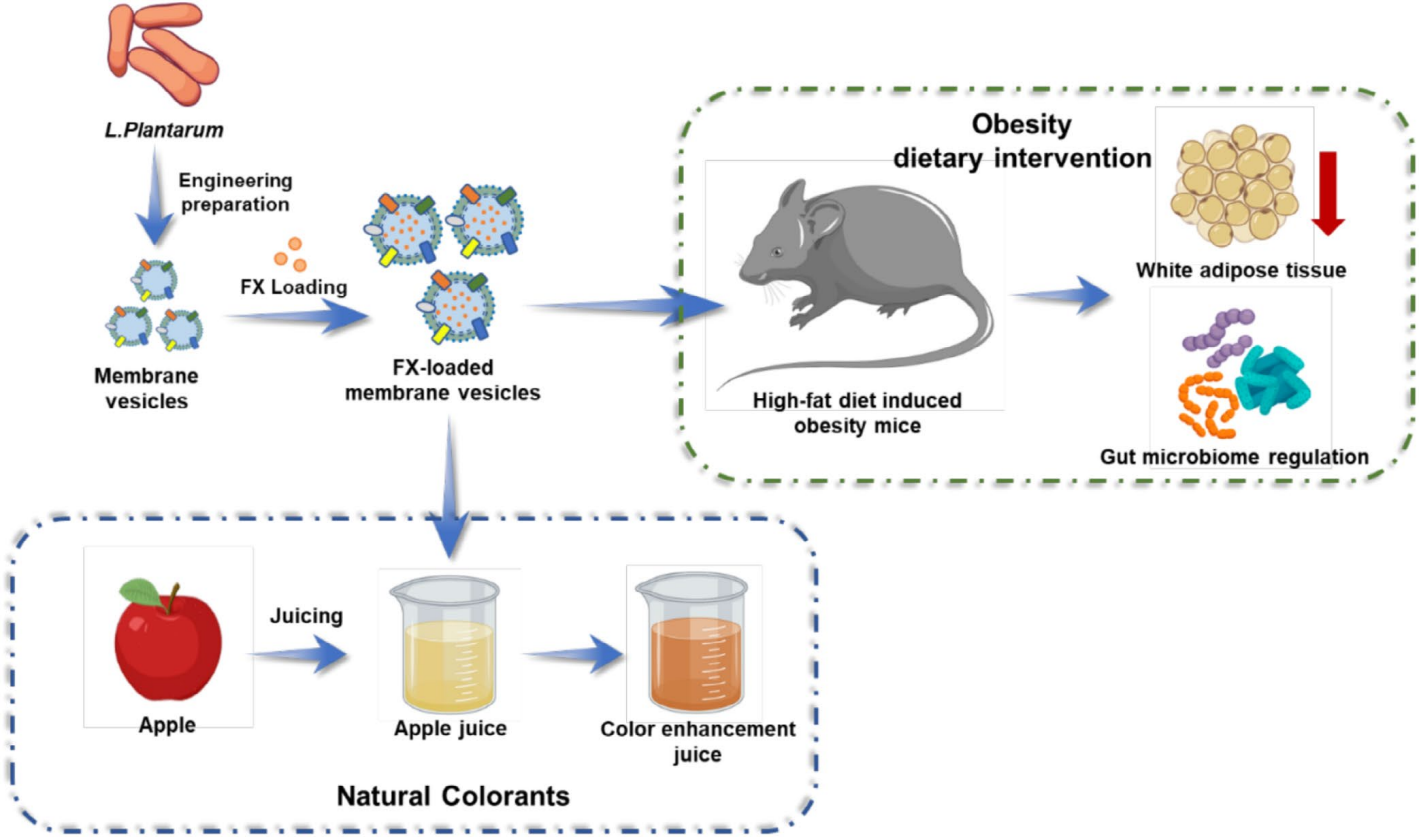
Schematic diagram of the fabrication of FX‐MVs and its application in nutritional intervention for high‐fat diet induced obesity and apple juice as natural colorant.

In this study, we developed fucoxanthin‐loaded probiotic membrane vesicles (FX‐MVs) and further investigated their nutritional intervention effects in high‐fat diet‐induced obese mice. Additionally, we explored the potential of FX‐MVs as a natural colorant in fruit juices (Figure 1). The anti‐obesity effects of FX‐MVs were evaluated using animal models of high‐fat diet induced obese mice. Key parameters including body mass, plasma lipid levels, histological assessments, and gut microbiota composition were analyzed to demonstrate the efficacy of FX‐MVs in obesity intervention. Additionally, the impact of FX‐MVs as a natural colorant on fruit juice was assessed through colorimetric and sensory analyses. This study provides a theoretical foundation for the efficient delivery of marine bioactive compounds using probiotic membrane vesicles as nutritional interventions against obesity, and expands their application in real food systems.

## 2 Materials and Methods

### 2.1 Materials

The bacterial strain *Lactobacillus plantarum* used in this study was a laboratory‐preserved culture originally isolated from traditional fermented vegetables (Sichuan pickles, China). Fucoxanthin (purity ≥ 75%) was commercially obtained from Shandong Jiejing Group Co. Ltd. (Shandong, China). MRS broth was procured from Guangdong Huankai Microbial SCI&Tech Co. Ltd. (Guangzhou, China). Fresh apples (Red Fuji) were acquired from a local agricultural market (Jianong Market, Xiamen, China). All chemical reagents employed in this investigation were of either chromatographic grade (HPLC) or analytical grade (AR), as specified in respective experimental protocols.

### 2.2 Preparation of Probiotic Synthetic Membrane Vesicles

The probiotic synthetic membrane vesicles (SyMVs) were prepared using a previously reported method (Liang, Liu, Li, Wu, et al. 2023). In brief, *Lactobacillus plantarum* was cultured in MRS broth at 37°C until mid‐logarithmic phase. Bacterial cells were harvested by centrifugation (8000 ***g***, 10 min, 4°C) and washed twice with sterile phosphate‐buffered saline (PBS, 0.01 M, pH 7.4). Subsequently, the bacterial cells were treated with lysozyme at 37°C for 24 h with gentle agitation. The protoplasts were precipitated via centrifugation (3500 ***g***, 10 min, 4°C), resuspended in ice‐cold PBS, and disrupted by probe ultrasonication (Scientz JY92‐IIDN homogenizer, China) for 30 min in an ice‐water bath. Cellular debris was sequentially removed through differential centrifugation (3500 ***g***, 10 min; 12,000 ***g***, 20 min). The clarified supernatant was ultracentrifuged at 100,000 ***g*** for 1 h and the membrane fragments were collected, which were subsequently redispersed in sterile PBS and sonicated in ice‐cooled bath (Shumei KQ5200DE, China) for 30 min. The resulting SyMVs were cryopreserved at −80°C for downstream applications.

### 2.3 Preparation and Purification of Fucoxanthin‐Loaded Probiotic Membrane Vesicles

The fucoxanthin‐loaded probiotic membrane vesicles (FX‐MVs) were prepared using a previously reported method (Liang, Liu, Li, Wu, et al. 2023). The fucoxanthin‐loaded probiotic membrane vesicles were prepared by co‐incubation. In brief, the protoplast‐derived fragments obtained through ultrasonic disruption (as described in Section 2.2) were combined with FX (dissolved in anhydrous ethanol) at a mass ratio of 4:1. The resulting mixture was subjected to ultrasonic treatment in an ice‐cooled bath for 30 min to facilitate integration. Ethanol was subsequently removed from the system via rotary evaporation at 37°C under reduced pressure. The suspension was centrifuged at 10,000 ***g*** for 15 min at 4°C to remove unencapsulated FX, and the supernatant was subsequently collected. The clarified supernatant was ultracentrifuged at 100,000 ***g*** for 1 h. The resulting pellets were resuspended in sterile PBS and cryopreserved at −80°C for further use.

### 2.4 Characterization of SyMVs and FX‐MVs

The characterization of SyMVs and FX‐MVs was to refer to the methods described previously with some modifications (Liang et al. 2025; Wu et al. 2024; Zhong et al. 2025). The size and morphology of samples were determined and observed by dynamic light scattering (DLS) instrument (LS Instruments AG, Switzerland) and Transmission Electron Microscopy (Hitachi HT‐7700, Japan), respectively.

The encapsulation efficiency (EE) and the loading capacity (LC) of FX‐MVs were determined according to previous methods (Liang, Liu, Li, Li, et al. 2023). Briefly, during the preparation of FX‐MVs, the mass of added FX was recorded. Subsequently, the obtained FX‐MVs were dissolved in PBS solution, and the absorbance at 446 nm was recorded by an HT microplate reader. The amount of protein was measured by the BCA protein quantification kit. The EE was calculated with the following equation:

$$\mathrm{EE}\;\left(\%\right)=M_{f}/\mathrm{FX}\;\text{added}\times 100\%$$

The LC was calculated with the below equation:

$$\mathrm{LC}\;\left(\%\right)=M_{f}/M_{\text{prot}}\times 100\%$$

Here, M_f_ is the mass of FX in the FX‐MVs and M_prot_ is the protein content of the FX‐MVs.

### 2.5 Intervention in High‐Fat Diet Induced Mice Obese

The entire animal experiment was followed the regulations of the Jimei University Science and Technology Ethics Committee (No. 20250401866). Fifty male C57BL/6 mice were obtained from the Xiamen University Laboratory Animal Center (Xiamen, China). All experimental mice were maintained in an individual ventilated caging (IVC) system under standard lighting conditions, with the temperature and humidity controlled at 21°C–24°C and 60%–65%, respectively. Upon arrival, all mice were acclimated to the environment for 7 days before the experiment. C57BL/6 mice were randomly assigned to five groups (*n* = 10). Mice in the control group were fed a standard chow diet (Sipeifu Biotechnology Co. Ltd., SPF‐F02) and received intragastric administration of normal saline once daily. Mice in the model group were fed a high‐fat diet (containing 60% fat, Xietong Medicine Bioengineering Co. Ltd., XTHF60) and also received intragastric administration of normal saline once daily. Mice in the FX, SyMVs, and FX‐MVs groups were fed a high‐fat diet and received intragastric administration of FX, SyMVs, or FX‐MVs, respectively, once daily. The intervention dose of the FX group was 15 mg/kg mice body weight. The relative content of the SyMVs group and the FX‐MVs group was calculated based on the loading capacity to ensure that the intervention dose was consistent among all groups. During the experiment, mice had ad libitum access to food and water, and their body weights were measured weekly.

After the 8‐week intervention, all mice were subjected to a 12‐h fasting period, and blood samples were collected via orbital bleeding. The blood samples were centrifuged at 3000 ***g*** for 15 min at 4°C to separate the serum, which was subsequently stored at −80°C for further testing. After the mice were sacrificed under anesthesia, the liver and white adipose tissue (WAT) were excised, photographed, weighed, and stored at −80°C for subsequent analyses. Additionally, intestinal contents of mice in each group were harvested and stored at −80°C for further investigation.

The level of total cholesterol (TC) and triglyceride (TG) inside the liver and the high‐density lipoprotein cholesterol (HDL‐C), low‐density lipoprotein cholesterol (LDL‐C), alanine aminotransferase (ALT), and aspartate aminotransferase (AST) in serum were evaluated using commercial kits (Nanjing Jiancheng Bioengineering Institute, China) according to the instructions. The WAT was collected and fixed in formalin solution (4%, w/v). The fixed tissues were embedded in paraffin and stained with hematoxylin and eosin (H&E) and observed with an optical microscope. The gut microbiota in mouse intestinal content was determined by the V3‐V4 region of 16 S rRNA sequencing. The samples were analyzed through the processes of genomic DNA extraction, PCR amplification, fluorescence quantification, library building, and NovaSeq sequencing. The process was completed in Shanghai BioTree Biomedical Technology Co. Ltd. (Shanghai, China). Gut microbiota composition, α‐diversity, and species abundant analysis were performed on the cloud platform.

### 2.6 Preparation of Fresh Apple Juice and FX‐MVs Fortified Apple Juice

The preparation of fresh apple juice was to refer to the methods described previously with some modifications (Zhong et al. 2024). Fresh apples (250 g) were peeled, combined with 1 L of deionized water, and homogenized using a commercial juicer. The resulting mixture was subsequently subjected to centrifugation followed by filtration to remove pulp residues and impurities, yielding fresh apple juice (AJ). FX‐MVs were subsequently mixed into the apple juice in the proportion of 5 g of FX/L apple juice, resulting in the production of FX‐MVs fortified apple juice (FX‐MVs@AJ).

### 2.7 Color Analysis

Chromatic properties were analyzed using an adapted image processing method (Velasco‐Pérez and Ramos‐Escudero 2024). Sample images were acquired with a smartphone camera with a resolution of 12 megapixels (Apple Inc., USA) under natural light conditions on a white background (standard mode, without additional color calibration). The acquired images were subsequently processed using ImageJ software (version 1.53e, National Institutes of Health, USA). A 100 × 100 square area from the middle of the picture was selected to conduct histogram analysis to extract the RGB parameters of the predefined area.

Colorimetric quantification was further performed using a portable grating spectrophotometer (YS3010, 3NH Technology Co. Ltd., China). Prior to analysis, the instrument was calibrated using standard black and white reference tiles. Triplicate measurements of the CIELAB color coordinates (L*, a*, b*) were recorded for each sample. Derived chromaticity parameters, including chroma (C*ab) and total color difference (ΔE), were calculated according to established equations (Jafari et al. 2020):

$$C^{*}\mathrm{ab}=\sqrt{a^{*2}+b^{*2}}$$

$$\mathrm{\Delta E}=\sqrt{{\left(L_{1}^{*}-L_{2}^{*}\right)}^{2}+{\left(a_{1}^{*}-a_{2}^{*}\right)}^{2}+{\left(b_{1}^{*}-b_{2}^{*}\right)}^{2}}$$

where $L_{2}^{*}$, $a_{2}^{*}$ and $b_{2}^{*}$ represented L* value, a* value and b* value of FX‐MVs@AJ; $L_{1}^{*}$, $a_{1}^{*}$ and $b_{1}^{*}$ represented L* value, a* value and b* value of fresh AJ.

### 2.8 Analysis of Olfactory and Gustatory Characteristics

The analysis of olfactory and gustatory characteristics were to refer to the method described previously (Liang et al. 2025). The volatile components of the samples were analyzed using a portable PEN3 E‐nose system (Airsense PEN3, Germany). The device is equipped with an array of 10 gas sensors (Table S1). Experimental procedures were as follows: 2 mL of the sample was precisely aliquoted into a dedicated headspace vial and subjected to 30 min of static headspace enrichment. Direct headspace injection mode was employed for detection. Instrumental parameters were set as follows: sampling interval of 1 s/group, sensor self‐cleaning time of 100 s, sample pretreatment time of 5 s, carrier gas flow rate of 400 mL/min, and analysis acquisition duration of 100 s. Data from the stable response interval (69–71 s) were selected for subsequent analysis.

The taste profiles of the samples were evaluated using an SA402B E‐tongue system (Insent, Japan), which integrates six specific taste sensors (Table S2). Prior to testing, samples were thoroughly vortexed. The detection protocol included the following steps: (1) sensor activation in a cleaning solution for 90 s; (2) equilibration in a reference solution (30 mM KCl +0.3 mM tartaric acid) for 120 s; (3) zero‐point calibration at the equilibrium position for 30 s; (4) sample measurement for 30 s to acquire initial taste values; and (5) post‐cleaning in fresh reference solution for 3 s, followed by another 30 s detection to obtain aftertaste values. All measurements were conducted under constant temperature conditions (25°C ± 0.5°C).

### 2.9 Quantification of FX

The quantification of FX was performed by High performance liquid chromatography (HPLC), referencing to previously published literature with some modifications (Salvia‐Trujillo et al. 2015). FX standard solution (0, 2.5, 5, 25, 50, 100 μg/mL) was prepared by dissolving FX in methanol. The samples (1 mL) were homogenized with 1 mL ethyl acetate/methanol (2:1) and centrifuged. The organic phase was dried under nitrogen, solubilized in 1 mL methanol, and subjected to HPLC after filtration. The FX concentration of FX was determined by theHPLC system (Unimicro EasySep‐3030, China). The samples and standards were separated on a C18 reverse phase column (Unimicro GS‐220‐5‐C18‐AP, 4.6 mm × 250 mm) with acetonitrile in methanol (850 mL acetonitrile/L mixed solution) at a flow rate of 1 mL/min. FX was detected at 450 nm.

### 2.10 Statistical Analysis

The experimental data were subjected to three repetitions. Statistical analysis was performed using PASW Statistics 18 software, with one‐way analysis of variance (ANOVA) and Duncan's multiple range test employed to evaluate significant differences among groups. Data were presented as means ± SD. A significance level of *p* < 0.05 was considered statistically significant and indicated by distinct lowercase letters.

## 3 Results and Discussion

### 3.1 Characterization of FX‐MVs

**FIGURE 2:**
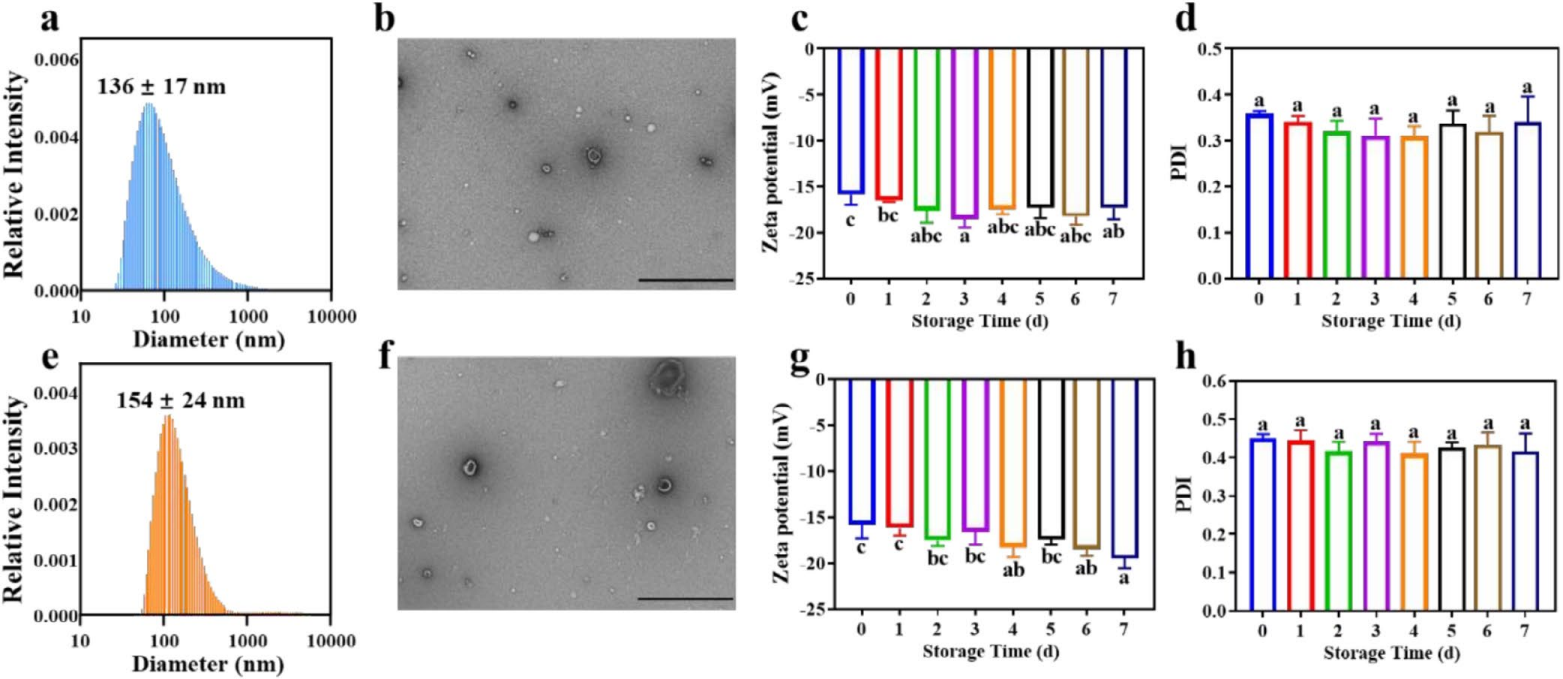
Characterization of SyMVs: (a) Size distribution and (b) TEM image; Changes of (c) zeta potential and (d) PDI in 7 days; Characterization of FX‐MVs: (e) Size distribution and (f) TEM image; Changes of (g) zeta potential and (h) PDI in 7 days; The scale bar is 1 μm.

Extracellular membrane vesicles are highly promising as carriers of active components owing to their excellent immunological compatibility and their unique ability to penetrate physiological barriers that cannot be achieved by synthetic delivery carriers (Herrmann et al. 2021; Liu et al. 2022; Reiner and Somoza 2019). In this study, we developed probiotic membrane vesicles as a novel encapsulation system for the efficient delivery of FX. The SyMVs and FX‐MVs were characterized using DLS and TEM. The results demonstrated that SyMVs exhibit a mean particle size of 136 ± 17 nm and possess a spherical nanoparticle morphology, displaying the characteristic vesicle shape with a smooth surface (Figure 2a,b). The initial zeta potential of SyMVs was −15.9 ± 1.1 mV and remained stable within the range of −16.5 to −18.5 mV over a 7‐day period (Figure 2c), consistent with findings reported in previous literature (Liang et al. 2025). The PDI value was 0.358 ± 0.006 and showed no significant variation during the same period (Figure 2d). Meanwhile, FX‐MVs exhibited a mean particle size of 154 ± 24 nm and showed a morphology similar to that of SyMVs (Figure 2e,f). Its initial zeta potential was −15.8 ± 1.4 mV, with a PDI of 0.451 ± 0.009, and exhibited a variation pattern similar to that of SyMVs (Figure 2g,h). In addition, the encapsulation efficiency and loading capacity of FX‐MVs were 87.14% and 53.64%, respectively.

### 3.2 Alleviating the Clinical Symptoms of High‐Fat Diet Induced Obese in Mice

**FIGURE 3:**
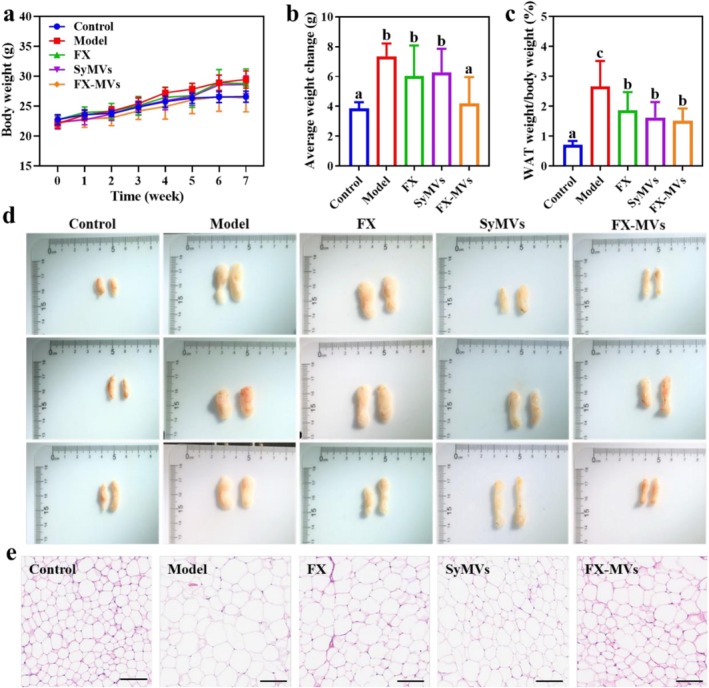
Effect of FX‐MVs intervention on HFD‐induced obesity in mice: (a) Body weight measurement; (b) Body weight change; (c) Ratio of white adipose tissue to body weight; (d) Photographs of WAT in different groups; and (e) H&E staining of mice WAT. Different letters represent significant differences from each other, *p* < 0.05.

Body weight is the most direct indicator for assessing individual obesity. To investigate the effect of FX‐MVs on obesity in high‐fat diet‐fed mice, body weight was measured weekly. As shown in Figure 3a, the body weight of mice in each group progressively increased over time. Notably, the weight gain in the model group was significantly higher compared to that in the normal control group. The weight changes of each group of mice after 7 weeks were further analyzed (Figure 3b). The results indicated that the average weight change was 3.85 ± 0.86 g in the Control group and 7.35 ± 0.86 g in the Model group. In comparison, the average weight change in the FX‐MVs group was 4.20 ± 1.76 g, which was most similar to that of the Control group. White adipose tissue (WAT) is crucial for maintaining whole‐body energy homeostasis and significantly influences metabolic and hormonal regulation (Heinonen et al. 2020). In healthy individuals, WAT undergoes controlled expansion and contraction to meet physiological demands. However, in pathological conditions such as obesity, WAT becomes dysfunctional, marked by excessive tissue expansion, disrupted lipid homeostasis, inflammation, hypoxia, and fibrosis (Solsona‐Vilarrasa and Vousden 2024). The proportion of white fat relative to body weight was compared across all groups (Figure 3c). The WAT weight/body weight of control group was 0.71% ± 0.13%. Compared to that of model group, FX‐MVs treatment groups showed a significant reduction in the WAT weight/body weight of mice, which indicated WAT overexpansion trend was markedly reversed with the additional FX‐MVs intervention. In the Figure 3d, the WAT morphology in the control group appeared normal with regular cell size and arrangement, whereas that in the model group exhibited significant enlargement. The intervention with FX‐MVs led to a notable improvement in the WAT morphology. Moreover, H&E staining demonstrated the impact of FX‐MVs on WAT in obese mice induced by a high‐fat diet (Figure 3e). The adipose cells in the control group were regularly arranged, small, and uniform. In contrast, the adipose cells in the model group exhibited significant enlargement. Compared to the model group, FX‐MVs treatment group resulted in a reduction in the average area of adipose tissue, with relatively normal intercellular substance.

### 3.3 Modulating Hepatic and Serum Lipid Metabolic Homeostasis

**FIGURE 4:**
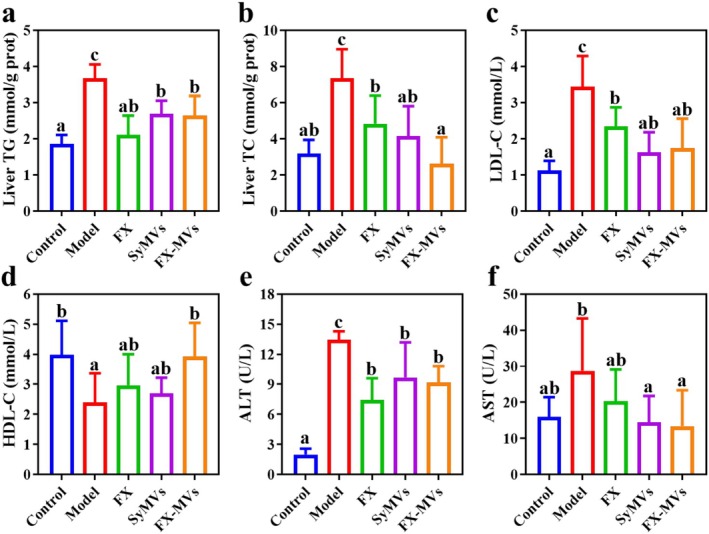
Contents of (a) TC and (b) TG in mice liver. Contents of (c) HDL‐C, (d) LDL‐C, (e) ALT, and (f) AST in mice serum (*n* = 6). Different letters in a column indicate significant difference (*p* < 0.05).

The liver plays a central role in individual lipid metabolism, encompassing key processes such as fat synthesis, degradation, transport, and cholesterol regulation (Li, Ren, et al. 2020; Li, Xu, et al. 2020). Therefore, additional investigations were conducted to examine certain indices of liver lipid metabolism. Figure 4a reveals that the TG level in the control group remained low, whereas the TG content in the liver of the model group was significantly elevated. The treatment with FX, SyMVs, and FX‐MVs respectively inhibited the increase in TG levels to varying degrees compared to the model group. Figure 4b illustrates the TC levels in the liver across different groups of mice, showing a consistent change trend that aligns with the observed patterns in liver TG level. Notably, the TC level in mice treated with FX‐MVs was decreased to a level comparable to that of the control group. Obesity is intricately linked to lipid metabolism disorders, with abnormal levels and functions of low‐density lipoprotein cholesterol (LDL‐C) and high‐density lipoprotein cholesterol (HDL‐C) serving as the central pathophysiological features. As depicted in Figure 4c,d, the LDL‐C level in the model group exhibited a marked abnormal increase, whereas the HDL‐C level demonstrated a significant reduction compared with the control group. Disruption of this balance constitutes a key manifestation of dyslipidemia, which may indicate underlying systemic metabolic abnormalities (Xu et al. 2024). After nutritional intervention with FX‐MVs, it can effectively restore this balance by increasing the level of HDL‐C and reducing the level of LDL‐C. Serum ALT and AST activities serve as reliable primary markers for detecting liver injury. The elevated levels of ALT and AST in the model group suggest that a high‐fat diet can induce liver damage in mice, while this damage is significantly alleviated by FX‐MVs treatment (Figure 4e,f). These results suggest that FX‐MVs have the potential to regulate lipid metabolism, maintain lipid homeostasis, and alleviate liver injury in high‐fat diet‐induced obesity mice.

### 3.4 Regulating the Composition of Gut Microbiota in Obesity

**FIGURE 5:**
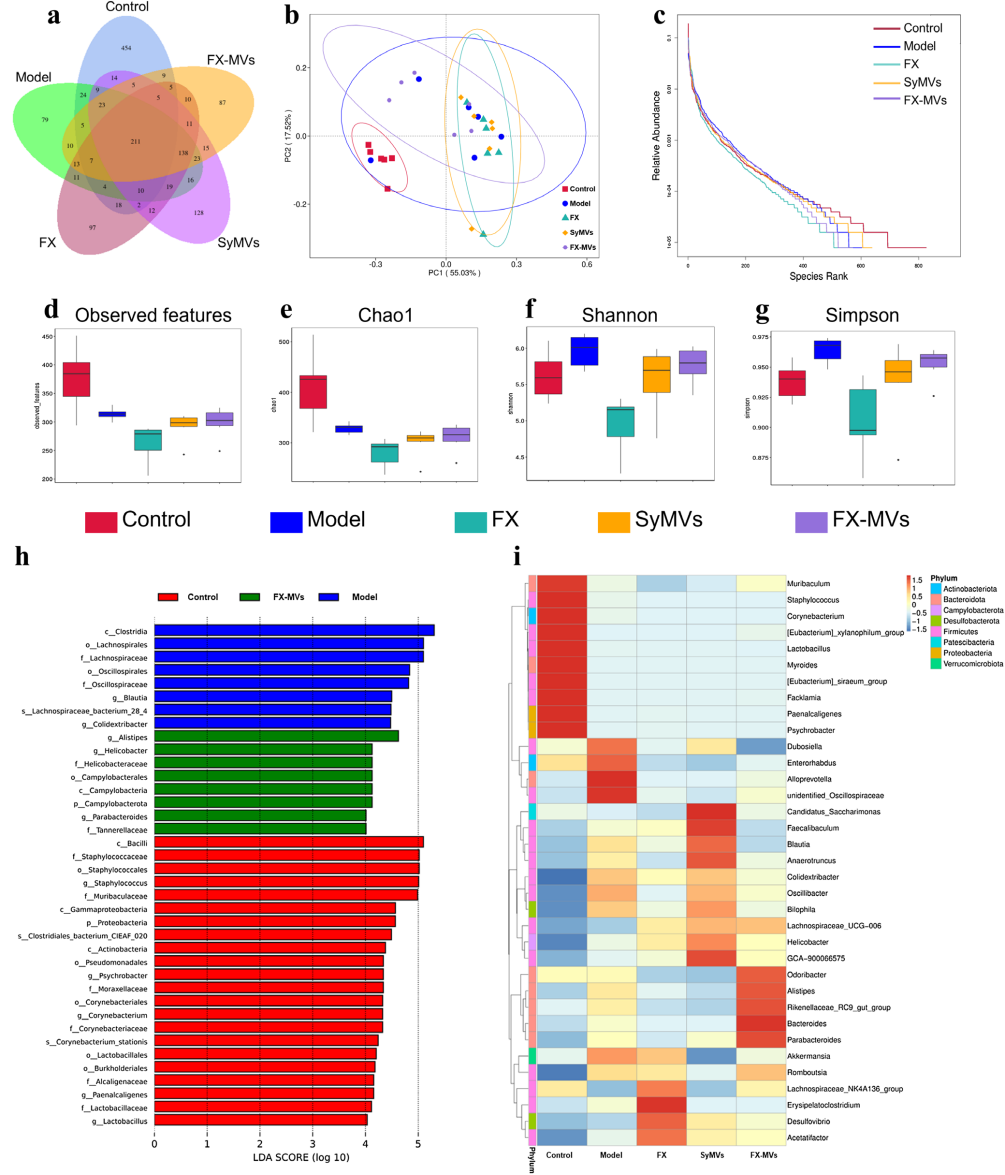
Diversity and composition analysis of gut microbiota upon treatment in HFD‐induced obesity mice: (a) Venn diagram analysis of the species composition; (b) principal coordinate analysis (PCoA) plots; (c) rank abundance curve; alpha‐diversity expressed by the (d) observed features, (e) Chao 1, (f) Shannon, and (g) Simpson; (h) LEfSe analysis; and (i) cluster heatmap at the genus level.

Many studies have demonstrated a strong association between high‐fat diet induced obesity and alterations in the gut microbiota (Cao et al. 2019; Luo et al. 2025; Wang et al. 2021). The composition of gut microbiota after FX‐MVs intervention was analyzed by sequencing of 16S rDNA in the V4 regions from mice fecal. The Venn diagram revealed that the number of shared operational taxonomic units (OTUs) across all groups was 211. Additionally, the numbers of unique OTUs in the control, model, FX, SyMVs, and FX‐MVs groups were 454, 79, 97, 128, and 87, respectively (Figure 5a). The principal coordinate analysis (PCoA) also revealed that the gut microbial structure of the five groups was different (Figure 5b). It is evident from the PoCA that the sample distances between the FX‐MVs treatment group and the control group were the smallest, indicating that their community structures were the most similar. Rank abundance was a way to assess species diversity, which could reflect the richness and uniformity of species. The control group exhibited the largest span on the horizontal axis, indicating the highest species richness. In comparison, the SyMVs, Model, FX‐MVS, and FX treatment groups showed a successive decrease in species richness (Figure 5c). The observed features and Chao1 index in the model group significantly decreased compared to the control group, while the levels in the SyMVs and FX‐MVs treatment groups were similar to those of the model group (Figure 5d,e). Moreover, a significant reduction in Shannon and Simpson indices was observed in the FX‐MVs group compared to the model group (Figure 5f,g). LEfSe analysis identified significant species differences between groups. At the genus level, *Blautia* and *Colidextribacter* were significantly enriched in the Model group, whereas *Alistipes* and *Parabaacteroides* were particularly abundant in the FX‐MVs group. Additionally, *Staphylococcus*, *Psychrobacter*, *Corynebacterium*, *Paenalcaligenes* and *Lactobacillus* were notably abundant in the Control group (Figure 5h). The species abundance clustering heatmap provided a more detailed visualization of the aggregation content in different treatment groups (Figure 5i).

### 3.5 Correlation Between Gut Microbiota Related to Obesity Key Indicators

**FIGURE 6:**
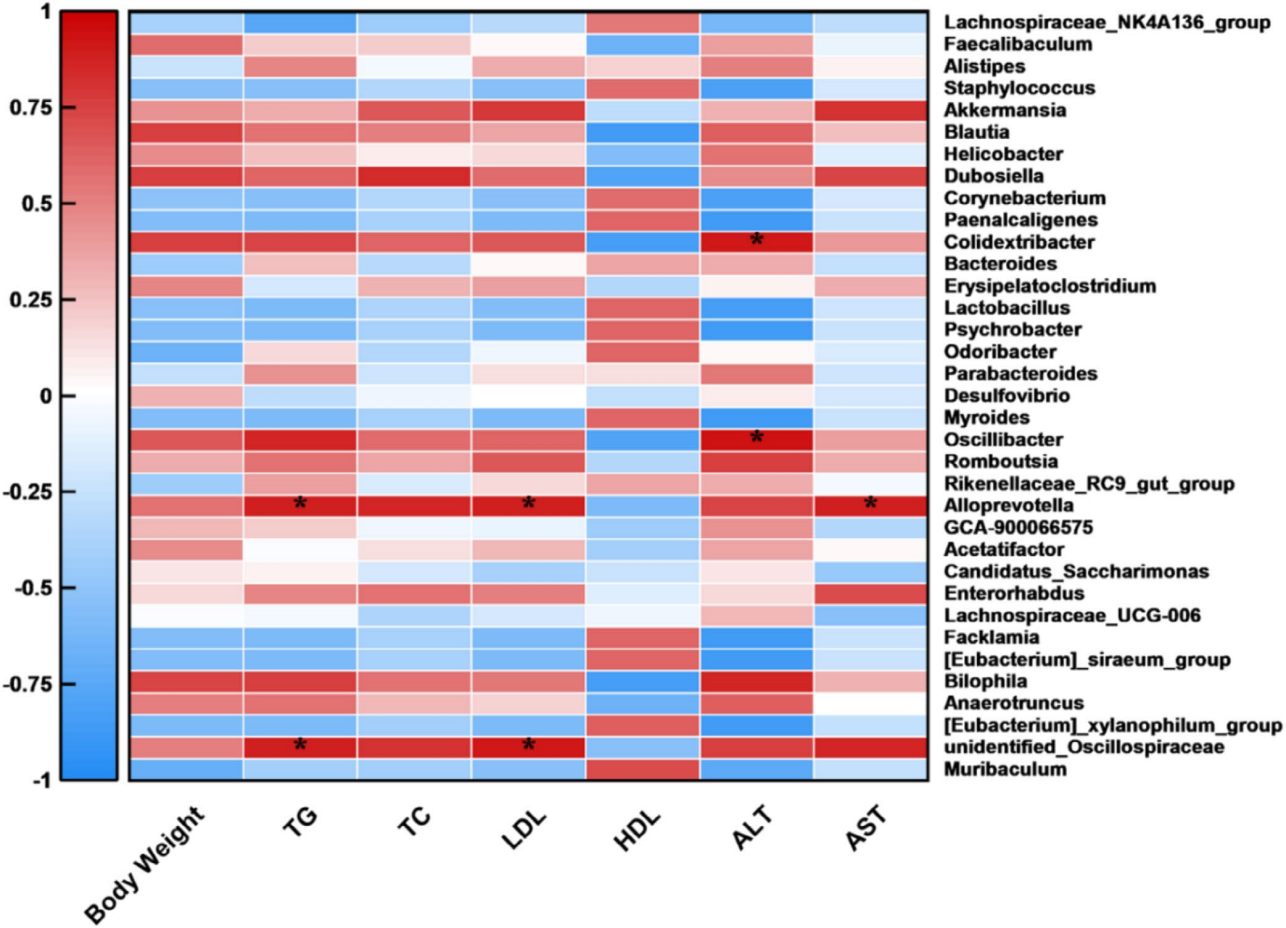
Correlation analysis between gut microbiota and obesity key indicators (*n* = 6).

To explore potential interaction pathways between the gut microbiota and liver and serum metabolic indices associated with obesity in mice, we calculated the Pearson correlation coefficients between differentially abundance liver and serum metabolic indices and differentially abundance OTUs at the genus level. As illustrated in Figure 6, correlation analysis revealed that *Colidextribacter* and *Oscillibacter* were positively associated with ALT levels (*p* < 0.05). Furthermore, *Alloprevotella* and *unidentified Oscillospiraceae* exhibited positive correlations with TG and LDL levels (*p* < 0.05). Meanwhile, *Alloprevotella* was also positively correlated with AST levels (*p* < 0.05). Noteworthily, The *Lachnospiraceae NK4A136 group*, *Staphylococcus*, *Corynebacterium*, *Paenalcaligenes*, *Lactobacillus*, *Psychrobacter*, *Odoribacter*, *Myroides*, *Facklamia*, *[Eubacterium] siraeum group*, [*Eubacterium*] *xylanophilum group*, and *Muribaculum* exhibit correlations with the healthier trends of liver and serum metabolic indicators in mice, which were worthy of attention as potential probiotics for combating obesity.

Short‐chain fatty acids (SCFAs), the primary metabolites derived from gut microbial fermentation of dietary fiber, have been extensively demonstrated to regulate host energy metabolism and inflammatory status through regulation of histone deacetylases (Luu et al. 2019). Correlation analysis revealed that the relative abundance of *Lactobacillus* was significantly negatively correlated with serum TC and LDL‐C levels and positively correlated with HDL‐C levels. Accumulating evidence indicates that *Lactobacillus* species exert anti‐obesity effects through multiple SCFA‐mediated pathways: they upregulate hepatic PPAR‐α and CPT‐1α expression, suppress lipogenesis via SREBP‐1c inhibition, and enhance mitochondrial β‐oxidation through GPR41/43 activation (Cui et al. 2025; Tang et al. 2022). Therefore, the FX‐MVs‐induced enrichment of SCFA‐producing bacteria likely contributes to the amelioration of dyslipidemia through a coordinated “microbiota–metabolite–liver” axis. In addition, a hallmark characteristic of high‐fat diet‐induced gut dysbiosis is the overgrowth of Gram‐negative opportunistic pathogens. This overgrowth leads to increased translocation of intestinal‐derived lipopolysaccharide (LPS), metabolic endotoxemia, and systemic low‐grade inflammation, thereby exacerbating obesity and hepatic steatosis (Nishimura et al. 2021; Park et al. 2024). FX‐MVs intervention significantly reduced the relative abundance of *Oscillibacter*, which exhibited a significant positive correlation with serum ALT and AST levels, suggesting that FX‐MVs may alleviate hepatic inflammatory injury by suppressing LPS‐producing bacteria. Furthermore, probiotic‐derived membrane vesicles have been demonstrated to upregulate the expression of intestinal tight junction proteins (Claudin‐1, Occludin), thereby restoring intestinal barrier function (Olovo et al. 2024).

Collectively, these findings clearly indicate that FX‐MVs improve metabolic disorders through a dual–synergistic mechanism. Firstly, they enrich the SCFA‐producing microbiota. By doing so, they promote fatty acid oxidation and inhibit fat synthesis via the “microbiota–metabolite–liver” axis, which in turn improves lipid profiles. Secondly, they suppress LPS‐producing opportunistic pathogens and enhance intestinal barrier function. This leads to a reduction in LPS translocation and liver inflammatory responses. This “reducing source and strengthening barrier” strategy acts in concert to alleviate obesity and steatosis, ultimately restoring metabolic homeostasis.

### 3.6 Enhancement of the Color Appearance of Fresh Apple Juice

Color is a critical parameter widely employed to assess food quality criteria, as it significantly influences consumer preferences for specific food products. In the health industry, there has been a growing trend toward utilizing natural pigments rather than artificial ones to enhance the color appearance of food, driven by consumer demand for healthier and more natural options. In this study, we further validated the potential application of FX‐MVs as a natural colorant in fresh apple juice by evaluating their color performance and flavor impact.

**FIGURE 7:**
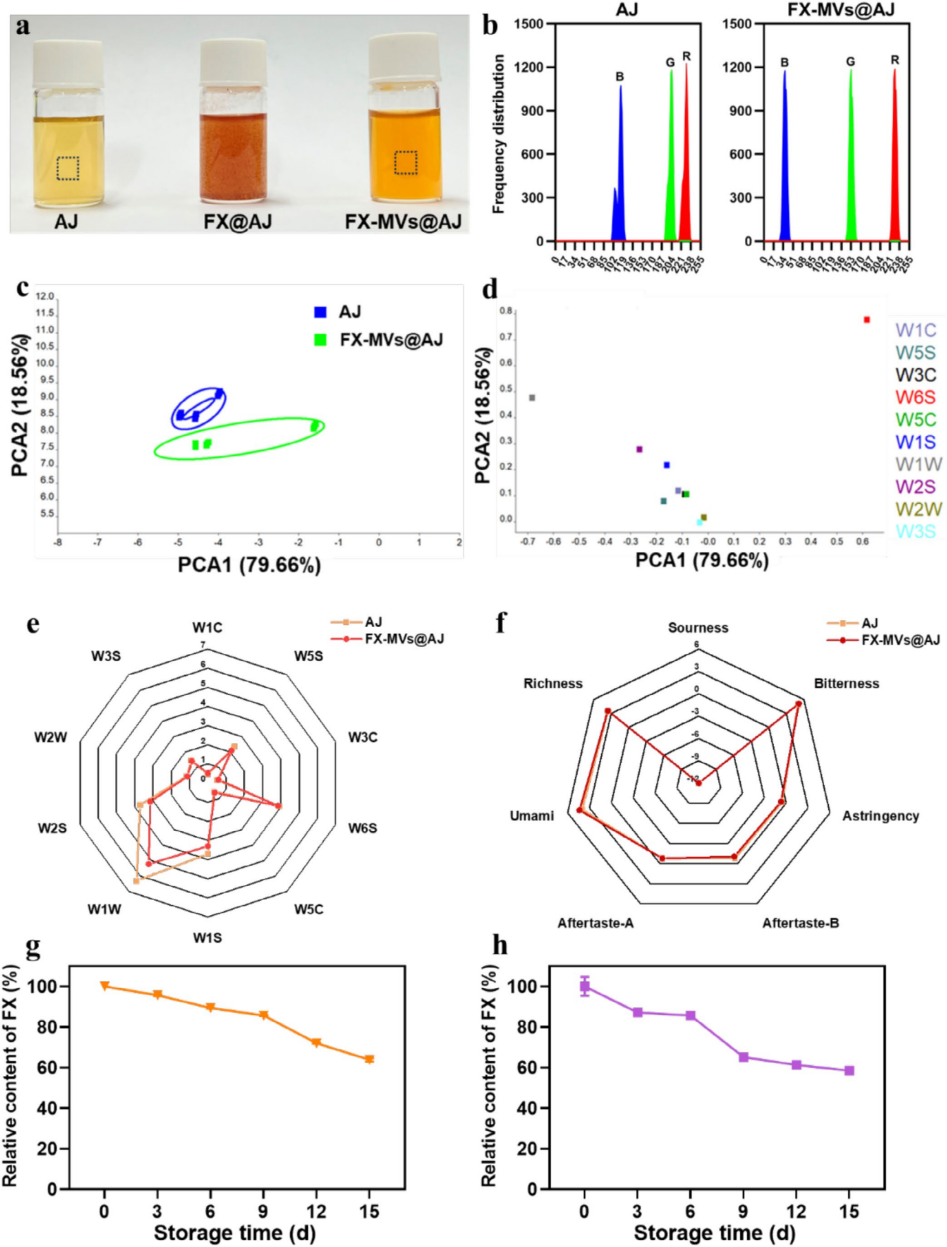
Application of FX‐MVs as natural colorant in fresh apple juice: (a) Photographs of different samples; (b) The color frequency distribution in red, green, and blue; (c) PCA analysis of E‐nose responses; (d) PCA analysis of E‐nose sensors; (e) Radar map of E‐nose sensors; (f) Radar map of E‐tongue; Relative content of FX in FX‐MVs@AJ at different storage temperature: (g) 4°C and (h) 25°C.

As depicted in Figure 7a, the fresh AJ had a pale orange hue. Upon the direct addition of FX to AJ, a hydrophobic suspension was formed, resulting in no alteration of AJ's color. In contrast, the introduction of FX‐MVs facilitated effective dispersion of FX within AJ, leading to substantial changes in its chromaticity, with the sample appearing as a darker orange‐yellow hue. Furthermore, the color channel composition of the samples was quantitatively analyzed via the image analysis software ImageJ, and the resulting data are presented in Figure 7b. The R channel color frequency distributions of the two samples were almost identical. Compared to fresh AJ, G and B channel color frequency decreased sharply, which suggested a tendency for FX‐MVs@AJ to exhibit yellowish hues. The color of apple juice with added FX‐MVs was quantified using a colorimeter. The visual appearance of the sample can be characterized by its L*a*b* values, where L* represents brightness, a* denotes the red‐green coordinate, and b* indicates the blue‐yellow coordinate, respectively (Liang et al. 2025). As shown in Table 1, the L*a*b* values of fresh AJ were 55.53 ± 0.41, −0.03 ± 0.08, and 23.17 ± 0.54, respectively. In contrast, the L* value for FX‐MVs@AJ was 51.36 ± 0.02, with the a* value of 6.48 ± 0.22 and the b* value of 35.01 ± 0.22. The ΔL*, Δa*, Δb* were 4.17 ± 0.42, 6.46 ± 0.21 and 11.23 ± 0.52, respectively. These results indicated that the addition of FX‐MVs resulted in a decrease in the brightness of the apple juice appearance and a tendency to yellow color, which was consistent with the color channel analysis.

**TABLE 1:** Color analysis of different sample.

Sample	L*	a*	b*	Δb*	C*ab	ΔE
AJ	55.53 ± 0.41a	−0.03 ± 0.08b	23.17 ± 0.54b	11.23 ± 0.52	23.17 ± 0.54b	13.62 ± 0.44
FX‐MVs@AJ	51.36 ± 0.02b	6.48 ± 0.22a	35.01 ± 0.22a		35.01 ± 0.48a	

*Note:* Data were presented as means ± SD and different letters represent significant differences from each other, *p* < 0.05.

The chromatic intensity (C*ab) and total color variation (ΔE) were quantitatively analyzed to evaluate color characteristics. Chroma values (C*ab) reflect color saturation intensity, where elevated magnitudes correspond to enhanced vividness. Comparative measurements revealed distinct chromatic profiles: fresh AJ exhibited a C*ab of 23.17 ± 0.54, while FX‐MVs@AJ demonstrated significantly higher saturation at 35.01 ± 0.48. This 51.1% increase in chroma values clearly demonstrates the successful color enhancement achieved through FX‐MVs modification.

For comprehensive color assessment, the ΔE metric was employed to quantify overall chromatic divergence between specimens. With a calculated ΔE value of 13.62 ± 0.44 between the two samples, this measurement substantially exceeds the established 3.5 threshold for human visual discrimination, which confirmed that FX‐MVs@AJ exhibits statistically significant and visually perceptible color alterations compared to that of fresh AJ (Athira et al. 2019). The aforementioned findings suggest that FX‐MVs can serve as a natural food colorant, imparting yellowish‐orange hues and thereby enhancing the chromatic attributes of the final product.

### 3.7 Effect on Flavor Perception of Fresh Apple Juice

The integration of chromatic additives into edible matrices requires rigorous preservation of native flavor characteristics while avoiding induction of extraneous olfactory or gustatory. Principal Component Analysis (PCA) was performed on E‐nose data obtained from fresh AJ and FX‐MVs@AJ samples, with results visualized in Figure 7c. Principal components PC1 and PC2 demonstrated individual contribution rates of 79.66% and 18.56%, respectively, achieving a cumulative variance explanation of over 98%. This statistical significance confirms the principal components' capacity to effectively differentiate between samples while maintaining representative integrity of the dataset. A minor divergence was observed in the PCA score plot between fresh AJ and FX‐MVs@AJ clusters, suggesting similar olfactory characteristics between the two samples. Sensor response analysis (Figure 7d) revealed that W6S (hydride) was the primary contributor to both principal components. Comparative analysis of volatile profiles via radar chart visualization (Figure 7e) revealed similar compositional patterns between the samples, with a notable reduction in response intensity observed for sensor W1W. Additionally, elevated response magnitudes from sensors W1W, W1S, W2S, and W6S indicated substantial contributions of sulfur‐containing compounds, aliphatic hydrocarbons, alcohols, and specific aromatic derivatives to the flavor profiles of both sample types. Although minor differences in sensor responses were observed (e.g., W1W), the overall flavor profile of FX‐MVs@AJ was still very close to that of fresh AJ. The addition of FX‐MVs did not cause significant deterioration of the flavor profile of fresh apple juice, and the main flavor characteristics were retained. E‐tongue evaluation of gustatory properties (Figure 7f) revealed consistent radar profiles among specimens, indicating limited taste differentiation. Quantitative taste parameter analysis highlighted umami, richness, and bitterness as the predominant sensory attributes in both samples. Collectively, these results indicate that incorporating FX‐MVs into fresh apple juice preserves its essential organoleptic properties and does not significantly alter its intrinsic flavor profile. Furthermore, the storage stability of FX in the final juice was evaluated. As shown in Figure 7g,h, the relative content of FX in FX‐MVs@AJ decreased over a 15‐day period under both cold storage (4°C) and room temperature (25°C). By the end of the storage period, the FX content in FX‐MVs@AJ retained 63% of its initial level under refrigeration, while it dropped to 58% at room temperature. Therefore, storing FX‐containing foods at low temperatures is recommended to minimize fucoxanthin degradation and maintain its biological activity.

## 4 Conclusion

This study demonstrated that probiotics membrane vesicles encapsulating fucoxanthin exhibit dual functionality as a therapeutic agent against diet‐induced obesity and a natural pigment for food applications. Experimental results revealed that FX‐MVs significantly alleviated HFD‐induced obesity by modulating lipid metabolism disorders and regulating the gut microbiota composition. In application studies, FX‐MVs enhanced the chromatic properties of fresh apple juice without causing significant deterioration of the flavor profile. These findings establish a mechanistic framework for using probiotic‐derived membrane vesicles as nanocarriers for bioactive compounds, highlighting their dual potential as both nutraceuticals and food‐grade colorants in functional food development. However, this study has several limitations. First, regarding the anti‐obesity mechanism of FX‐MVs, although we observed modulation of the gut microbiota and its correlation with improved metabolic parameters, key metabolites such as short‐chain fatty acids were not directly measured. Consequently, the proposed “dual‐synergistic mechanism” remains at a hypothetical level and requires confirmation through subsequent research. Second, in terms of food application, the efficacy of FX‐MVs as a natural colorant was validated only in a single matrix. Their performance in a broader range of food systems (e.g., beverages with varying pH values, dairy products) and under actual processing conditions (e.g., thermal treatment, pasteurization) was not investigated, limiting the generalizability of the conclusions. These limitations define this study as a proof‐of‐concept investigation, and future work should focus on deeper mechanistic elucidation and broader application validation to refine these findings. Future efforts could be directed toward the following two aspects: (i) Further confirming the causal role of the gut microbiota and deeply elucidate the anti‐obesity molecular mechanism by which FX‐MVs regulate the “microbiota‐metabolism‐organ” axis. (ii) Expanding the applicability of FX‐MVs across a broader range of food matrices, such as beverages with varying pH values, dairy products, and solid food systems, to validate their coloring performance and suitability, thereby comprehensively assessing the generalizability of this approach. (iii) Systematically evaluating the stability of FX‐MVs under typical food processing and storage conditions, including the effects of thermal treatment, light exposure, oxidative stress, and different temperature environments on their color attributes and retention of bioactive compounds.

## Author Contributions

**Duo Liang:** writing – original draft, visualization, software, funding acquisition, methodology, data curation, investigation, conceptualization. **Yueling Sun:** investigation, methodology. **Ritian Jin:** validation, writing – review and editing. **Ziling Liu:** methodology, investigation. **Rong Lin:** validation. **Jinfeng Wu:** methodology, investigation. **Shen Yang:** writing – review and editing, funding acquisition.

## Funding

This study was funded by the National Natural Science Foundation of China (32402010), the Natural Science Foundation of Fujian Province, China (2024J01107), and the Fujian Provincial Oceanic and Fisheries Bureau, China (FJHYF‐L‐2023‐17).

## Ethics Statement

All animal procedures were performed in accordance with the Guidelines for Care and Use of Laboratory Animals of Jimei University and approved by the Animal Ethics Committee of Jimei University (approval no. 20250401866).

## Conflicts of Interest

The authors declare no conflicts of interest.

## Supporting information


**Table S1:** Electronic nose sensor corresponding substance.
**Table S2:** Electronic tongue sensor corresponding taste.

## Data Availability

The data that support the findings of this study are available from the corresponding author upon reasonable request.
