# Supplementary material for: Application of Fucoxanthin‐Loaded Probiotic Membrane Vesicles in Dietary Intervention of High‐Fat Diet Induced Obese Mice and Color Improvement for Fruit Juice
Source: Food Sci Nutr. 2026 Mar 23;14(3):e71686. doi: 10.1002/fsn3.71686 (PMC13093531; doi:10.1002/fsn3.71686)
Supplement: Supplementary file 1 — Table S1: Electronic nose sensor corresponding substance. Table S2: Electronic tongue sensor corresponding taste. [file FSN3-14-e71686-s001.docx]

**Supplementary Materials**

**Application of fucoxanthin-loaded probiotic membrane vesicles in dietary intervention of** **high-fat diet induced obese mice and color improvement for fruit juice**

Duo Liang, Yueling Sun, Jinfeng Wu, Ziling Liu, Rong Lin, Ritian Jin, Shen Yang^*^

*College of Ocean Food and Biological Engineering, Jimei University, Xiamen 361021 Fujian, China*

*Corresponding Author:

Shen Yang, E-mail: yangshen@jmu.edu.cn.

Tel: +86-0592-6180638; Yindou Road 43, Jimei District, Xiamen 361021, China.

Table S1. Electronic nose sensor corresponding substance

| Sensor number | Sensor code | Substance |
| --- | --- | --- |
| 1 | W1C | aromatic compounds |
| 2 | W5S | nitrogen oxide |
| 3 | W3C | ammonia and aromatic compounds |
| 4 | W6S | hydride |
| 5 | W5C | Alkenes, aromatic groups, polar molecules |
| 6 | W1S | alkanes |
| 7 | W1W | sulfur compounds |
| 8 | W2S | alcohols and partially aromatic compounds |
| 9 | W2W | aromatic compounds and organic sulfur compounds |
| 10 | W3S | alkanes and aliphatic groups |

Table S2. Electronic tongue sensor corresponding taste

| Sensor number | Sensor | Taste information | |
| --- | --- | --- | --- |
|  |  | Initial taste | Aftertaste |
| 1 | C00 | Bitterness | - |
| 2 | AE1 | Astringency | - |
| 3 | CA0 | Sourness | - |
| 4 | CT0 | Saltness | - |
| 5 | AAE | Umami | Richness |
| 6 | GL1 | Sweetness | - |
